# Coupled Transmembrane Substrate Docking and Helical Unwinding in Intramembrane Proteolysis of Amyloid Precursor Protein

**DOI:** 10.1038/s41598-018-30015-6

**Published:** 2018-08-17

**Authors:** Nicolina Clemente, Alaa Abdine, Iban Ubarretxena-Belandia, Chunyu Wang

**Affiliations:** 1 Biochemistry and Biophysics Graduate Program, Center for Biotechnology and Interdisciplinary Studies, Rensselaer Polytechnic Institute, Troy, New York 12180 USA; 2 Department of Biological Sciences, Center for Biotechnology and Interdisciplinary Studies, Rensselaer Polytechnic Institute, Troy, New York 12180 USA; 3 Department of Pharmacological Sciences, Icahn School of Medicine at Mount Sinai, New York, NY 10029 USA; 4 Biofisika Institute (CSIC, UPV/EHU), Universidad del País Vasco (UPV/EHU), E-48940 Leioa, Spain

## Abstract

Intramembrane-cleaving proteases (I-CLiPs) play crucial roles in physiological and pathological processes, such as Alzheimer’s disease and cancer. However, the mechanisms of substrate recognition by I-CLiPs remain poorly understood. The aspartic I-CLiP presenilin is the catalytic subunit of the γ-secretase complex, which releases the amyloid-β peptides (Aβs) through intramembrane proteolysis of the transmembrane domain of the amyloid precursor protein (APPTM). Here we used solution NMR to probe substrate docking of APPTM to the presenilin homologs (PSHs) MCMJR1 and MAMRE50, which cleaved APPTM in the NMR tube. Chemical shift perturbation (CSP) showed juxtamembrane regions of APPTM mediate its docking to MCMJR1. Binding of the substrate to I-CLiP decreased the magnitude of amide proton chemical shifts δ_H_ at the C-terminal half of the substrate APPTM, indicating that the docking to the enzyme weakens helical hydrogen bonds and unwinds the substrate transmembrane helix around the initial ε-cleavage site. The APPTM V44M substitution linked to familial AD caused more CSP and helical unwinding around the ε-cleavage site. MAMRE50, which cleaved APPTM at a higher rate, also caused more CSP and helical unwinding in APPTM than MCMJR1. Our data suggest that docking of the substrate transmembrane helix and helical unwinding is coupled in intramembrane proteolysis and FAD mutation modifies enzyme/substrate interaction, providing novel insights into the mechanisms of I-CLiPs and AD drug discovery.

## Introduction

In intramembrane proteolysis (IP), an integral membrane protein is cleaved by intramembrane cleaving proteases (I-CLiPs)^1^ within the transmembrane domain (TM) to liberate biologically active fragments. As a unique form of signal transduction, I-CLiPs plays essential roles in numerous physiological processes such as embryonic development, immune responses and normal function of the nervous system. I-CLiPs also contribute to many diseases, including Alzheimer’s disease (AD) and cancer. γ-secretase, an aspartyl I-CLiP, cleaves within the transmembrane domain of APP (APPTM) to release amyloid-β peptide (Aβ), which aggregates to form senile plaque in the brain, a pathological hallmark of AD^2^. γ-secretase is a transmembrane protein complex whose catalytic component is the presenilin protein which harbors the active site aspartates^3–7^. Mutations in presenilin and APP (such as V44M in APPTM) can cause familial AD (FAD) characterized by early onset of dementia and increased Aβ42/Aβ40 ratio.

Unlike soluble proteases which recognize specific amino acid sequences, I-CLiPs display promiscuity against transmembrane substrates. To date, over 90 physiological substrates (e.g. Notch) are known for presenilin/γ-secretase, with no apparent consensus recognition motif ^8^. Substrate promiscuity of γ-secretase has contributed to the failure of clinical trials of γ-secretase inhibitors, e.g. through the inhibition of the Notch signaling pathway^9,10^. Thus understanding substrate/enzyme interaction in I-CLiPs will not only contribute to our fundamental understanding of I-CLiPs but also may provide novel insights for selective inhibition of γ-secretase in AD drug discovery.

**Figure 1:**
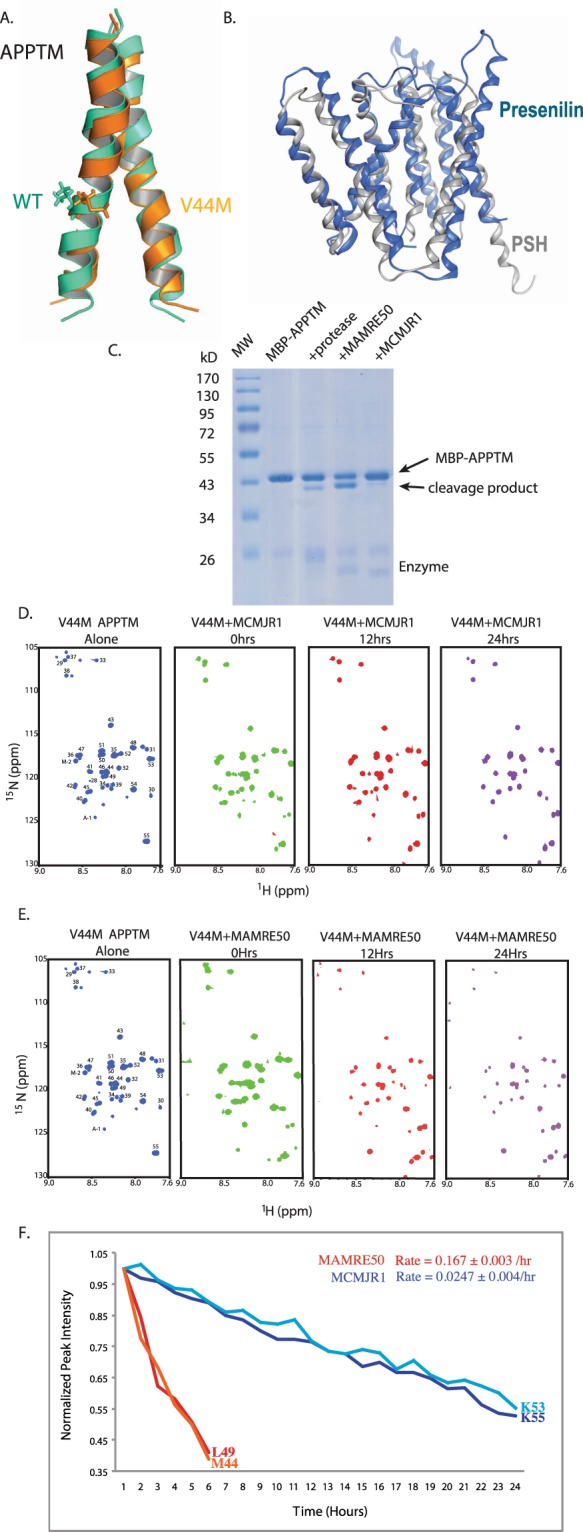
Intramembrane proteolysis of APPTM in solution NMR. (**A**) Overlaid NMR structures of WT (green) and FAD V44M (orange) APPTM, with residue 44 in stick mode. (**B**) Structure of PSH (grey) overlaid on presenilin (blue) with an RMSD of 3.1 Å. (**C**) Intramembrane proteolysis activity of MAMRE50, MCMJR1 and a control intramembrane protease against MBP-APPTM measured by SDS-PAGE after incubation at 37 °C for 12 hrs. Intramembrane proteolysis activity of MCMJR1 (**D**) and MAMRE50 (**E**) in solution NMR, evidenced by decreasing APPTM peak intensity and the appearance of sharp peaks in 2D ^15^N-^1^H HSQC at 40 °C over 24 hours, with enzyme:substrate at 1:1 molar ratio. Similar results were obtained for WT APPTM (data not shown). (**F**) Determination of the cleavage rate of APPTM from variation of peak intensity over time for select well-resolved peaks with high S/N.

Despite recent progress in the structure determination of I-CLiPs^11–15^, including human γ-secretase^4,16,17^ and the archaeal presenilin homologue (PSH) MCMJR1^11^, none of these structures contained a transmembrane substrate. Thus, how I-CLiPs recognize their transmembrane helical substrates remains a central, unresolved question in I-CLiP mechanism with important implications for AD drug discovery. Previously we have solved the NMR structure of the APPTM dimer (Fig. 1A) and characterized the structural effects of FAD mutations such as V44M^18^. V44M, an FAD mutation initially identified in French population (thus the name “French mutation”), causes dementia as early as forty years of age^19^. V44M increases Aβ42/Aβ40 ratio^19,20^, likely through enhancing the flexibility and accessibility of T48, the initial ε-cleavage site for Aβ42 production^18^. Here we used solution NMR to probe the interaction between APPTM and PSHs in intramembrane proteolysis and show that juxtamembrane residues in APPTM make initial contacts with PSH, and that unwinding of the substrate’s transmembrane helix is coupled with its recognition.

## Results and Discussion

### Intramembrane proteolysis of APPTM in the NMR tube

The PSH MCMJR1^21^ and its homolog MAMRE50 (45% sequence identity) from the archaeon Methanocella arvoryzae MRE50 were used as I-CLiPs in this study, while APPTM was used as substrate^22^. APPTM is a substrate for both γ-secretase^22^ and PSH^21,23^. PSH and γ-secretase share many biochemical and structural similarities. *In vitro*, the PSH MCMJR1 cleaves within APPTM of C99 to produce Aβs identical to those produced by the γ-secretase complex, and is inhibited by the same transition state analogs that target the presenilin active site^21,23^. In addition, the effect of FAD mutations in presenilin on Aβ42/40 ratio can be reproduced by analogous mutations in MCMJR1^23^. Finally, the crystal structures^11^ of MCMJR1 and human presenilin/γ-secretase (Fig. 1B)^16^ are similar with RMSD of 3.1 Å, and even the catalytic aspartates are in near perfect registry for both I-CLiPs.

APPTM and PSHs were purified as previously described^21,24,25^. In an established gel-based intramembrane proteolysis assay (Fig. 1C)^21^, both MCMJR1 and NAMRE50 cleaved within APPTM that was fused to maltose binding protein (MBP), with MAMRE50 demonstrating significantly higher enzymatic activity (Fig. 1C).

Next we measured intramembrane proteolysis of APPTM by solution NMR. Over 24 hours at 40 °C in an NMR sample of ^15^N-labeled APPTM and unlabeled MCMJR1 in 5% DPC micelles, APPTM peaks became progressively weaker, while new sharp peaks appeared between 122–130ppm (Fig. 1D). The absence of I-CLiP or the addition of the γ-secretase inhibitor III-31-C^26^ in the presence of MCMJR1 did not elicit any peak intensity changes, nor the appearance of new resonances in APPTM over the same time period (data not shown). When ^15^N-labeled APPTM was mixed with MAMRE50, APPTM peak intensity decreased faster while new sharp peaks appeared more rapidly (Fig. 1E). Fitting the NMR peak intensity changes to an exponential decay yielded rates of 0.167 ± 0.003 hr^−1^ and 0.0247 ± 0.0004 hr^−1^, for MAMRE50 and MCMJR1, respectively (Fig. 1F). Consistent with the gel-based intramembrane proteolysis assay, MAMRE50 catalyzed the cleavage of APPTM ~7 times faster than MCMJR1. To our knowledge, this is the first time that I-CLiP activity is directly observed with solution NMR, paving the way for studying the mechanisms of intramembrane proteolysis at atomic resolution.

### Juxtamembrane residues in APPTM participate in initial docking to MCMJR1

**Figure 2:**
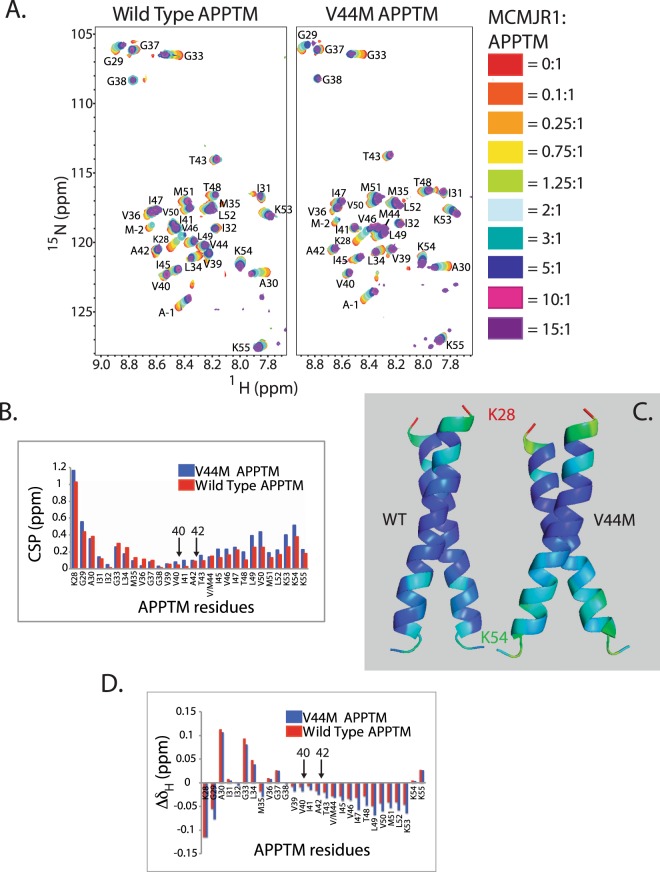
Solution NMR mapping of APPTM interaction with MCMJR1. (**A**) MCMJR1 titration into ^15^N labeled WT and V44M-APPTM at enzyme:substrate molar ratios from 0:1 to 15:1. (**B**) Combined ^15^N and ^1^H CSP vs. residue number for WT-APPTM (red) and V44M-APPTM (blue) at an enzyme:substrate molar ratio of 10:1. The largest combined CSPs were observed at the juxtamembrane region of APPTM. V44M experienced more combined CSP than WT, especially near the C-terminus. (**C**) Combined CSP mapped onto the structures of WT and V44M, colored from red (largest combined CSP) to blue (smallest combined CSP) in a rainbow color gradient. (**D**) Amide hydrogen chemical shift perturbation (Δδ_H_) at 10:1 molar ratio of MCMJR1 to APPTM for both WT-APPTM (red) and V44M-APPTM (blue). The data show a pattern of decreasing amide proton chemical shift at the C-terminal half of APPTM, indicating decreasing helical hydrogen bond strength and helical unwinding in the substrate. More unwinding was observed for the FAD mutant V44M than WT. Cut sites for Aβ40 and Aβ42 generation are indicated by arrows in (**B**) and (**D**).

It has been proposed in literature that substrates bind to I-CLiPs in two stages: first the substrate docks to an exosite which is distinct from the active site; then the substrate translocates from the exosite site to the active site^27–29^. We titrated unlabeled MCMJR1 into ^15^N-labeled APPTM WT and the V44M mutant (Fig. 2A). Little intramembrane proteolysis product was observed until the MCMJR1 to APPTM molar ratio reached 15:1, providing a window for probing substrate/I-CLiP interactions. Here we probed the interaction of APPTM with the substrate docking site on the enzyme, not with the active site, because the addition of an active site inhibitor L685,458 had no effect on the titration (data not shown).

Combined ^15^N and ^1^H chemical shift perturbations (combined CSP) were used here to probe the docking sites in APPTM and was calculated as $CSP=\sqrt{{(10{\rm{\Delta }}{\delta }_{H})}^{2}+{({\rm{\Delta }}{\delta }_{N})}^{2}}$, where Δδ_H_ and Δδ_N_ denote the change in chemical shift in the proton and nitrogen dimensions, respectively. In the presence of MCMJR1, combined CSPs were most prominent for residues close to the membrane-water interface of both WT and V44M APPTM (Fig. 2B,C). The largest combined CSP was observed at the N-terminal juxtamembrane residue K28. When the extent of presenilin-NTF binding to C99 was measured by photo-affinity mapping^27^, there is also a gradual increase in extent of binding from the TM center towards K28, mirroring the CSP pattern observed here. There is minimal interaction at the center of APPTM from V36 to A42, again consistent with the photo-affinity mapping. A gradual increase in CSP was observed towards the C-terminal part of TM, with largest perturbations centered at C-terminal lysine cluster (K53-K55) and near the ε-cleavage sites T48 and L49, where the initial cleavage by presenilin occurs^30^. Our CSP data are consistent with previous work on the mutagenesis of these juxtamembrane residues^31–33^ and suggest that the juxtamembrane residues in APPTM may initiate substrate docking, most likely through interaction with TM linker residues in PSH and γ-secretase. Although existence of APPTM as a dimer has been supported by numerous studies^34–39^, it is not known whether APPTM interacts with the enzyme as a dimer or monomer. Here, based on the lack of CSP at the dimer interface (Fig. 2B,C), APPTM likely docks to the enzyme as a dimer.

#### Effect of FAD mutation V44M on substrate docking

In the presence of MCMJR1, V44M mutation enhanced CSP towards the C-terminal end compared to APPTM WT (Fig. 2B,C). This observation suggests that this FAD mutation not only changes substrate conformation and dynamics as shown by us recently^18^, they also change the initial interaction with the I-CLiP.

### Helical unwinding of APPTM by MCMJR1 probed by changes in amide proton chemical shifts

The relationship between amide proton chemical shift and backbone hydrogen bonding in proteins has been well established^40–42^, with smaller chemical shifts corresponding to longer and weaker amide hydrogen bonds. Upon binding to MCMJR1, both WT APPTM and the V44M mutant displayed decreasing amide proton chemical shift at the C-terminal half, with a larger decrease towards the C-terminus of the TM (Fig. 2D). These chemical shift data indicate that the backbone hydrogen bonds at the the C-terminal region of APPTM are being weakened upon docking to the enzyme, indicating the unwinding of α-helical geometry in the region harboring the initial cleavage sites.

Although soluble proteases almost universally bind substrates within extended regions, which favor the complementary interactions that govern substrate specificity and facilitate access to the scissile peptide bond^43,44^, I-CLiPs bind transmembrane substrates that are presumably in α- helical conformation. This contrast has led to the hypothesis that transmembrane substrate helical unwinding is necessary for intramembrane proteolysis^45,46^. Our data lend support to this hypothesis and is consistent with a recent deep UV-Raman spectroscopy study which has shown that binding of *E. coli* rhomboid and MCMJR1 to the established I-CLiP substrate Gurken resulted in local unwinding of the transmembrane helix for cleavage^47^. It is likely that in intramembrane proteases the active site can not bind to a fully helical substrate and requires the helical unwinding at the docking site before the substrate can access the active site. Our data here demonstrate here that helical unwinding indeed occurs upon substrate docking to the the enzyme, priming its recognition and cleavage at the active site.

Interestingly, MCMJR1 binding caused larger decrease in C-terminal amide proton chemical shift in the FAD mutant V44M than in the WT (Fig. 2C). This is consistent with our previous finding that the V44M FAD mutation weakens helical hydrogen bonds at the C-terminal half of APPTM^18^, which are then more susceptible to unwinding by the enzyme than the WT. Together, our data demonstrate that the V44M FAD mutation not only affects the initial recognition of substrate in intramembrane proteolysis but also the concomitant unwinding around the cleavage site in the substrate.

### MAMRE50 binding causes larger CSP and more helical unwinding in APPTM

**Figure 3:**
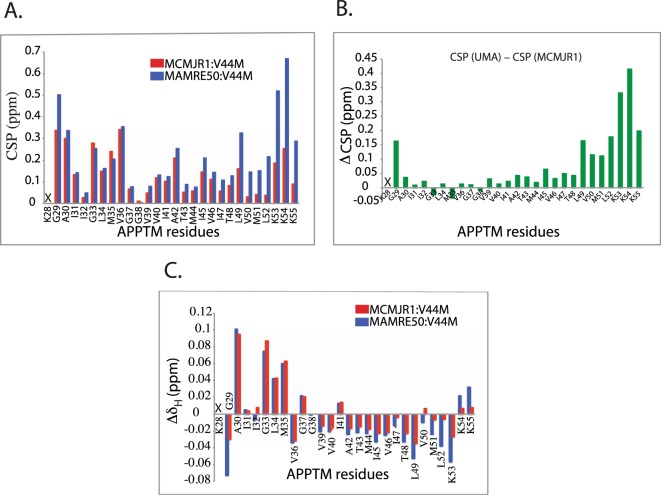
MAMRE50 caused more CSP and helical unwinding in APPTM than MCMJR1. (**A**) Combined CSPs in V44M-APPTM due to MAMRE50 (blue) and MCMJR1 (red) at at an enzyme to substrate molar ratio of 1:1. (**B**) CSP difference (ΔCSP) plot between CSP caused by MAMRE50 and that caused by MCMJR1 at an enzyme to substrate molar ratio of 1:1. (**C**) Change in amide proton chemical shift (Δδ_H_) in V44M-APPTM due to MAMRE50 (blue) and MCMJR1 (red). X indicates missing data.

To correlate I-CLiP activity with substrate docking, we also studied APPTM interaction with a higher activity PSH MAMRE50 (Fig. 1C). In order to minimize the effect of cleavage, we only used a PSH:APPTM ratio of 1:1 here, and the NMR spectrum was recorded immediately after the addition of enzyme. During this “dead period”, over 80% of APPTM remained uncleaved. As shown in Fig. 3A, MAMRE50 overall caused a similar combined CSP pattern as MCMJR1. However, there is a clear increase in the magnitude of the combined CSP (Fig. 3B) and a more prominent decrease in amide proton chemical shift in the C-terminal half of APPTM (Fig. 3C). These data suggest that a stronger initial docking, coupled with more helical unwinding of the substrate, contributes to the higher activity of MAMRE50.

## Conclusion

Based on chemical shift perturbation, docking of the substrate APPTM to the PSHs (MCMJR1 and MAMRE50) involves juxtamembrane residues and is coupled to unwinding of the transmembrane helix around the ε-cleavage site, priming the substrate for intramembrane proteolysis. Unwinding of the helical geometry around the cleavage site upon docking to the I-CLiP would favor the extended β-strand conformation that binds productively to the active site of proteases^43^. Our study represents the first high-resolution mapping of substrate-enzyme interaction in intramembrane proteolysis by solution NMR, providing novel insights into the mechanism of I-CLiPs and AD drug discovery.

## Methods

### Presenilin homolog (PSH) expression and purification

MAMRE50 and MCMJR1 enzymes were expressed and purified using the protocol in *J.W. Cooley et al*.^25^.

### Presenilin homolog cleavage assay

MCMJR1 and MAMRE50 enzymes were buffer exchanged into reaction buffer containing 20 mM Na-HEPES, 200 mM NaCl, 0.1% DDM at a pH 7 and were further diluted to a final concentration of 1 μM. The reactions were composed of substrate to enzyme at a molar ratio of 1.5:1 and were incubated for 12 hours at 37 °C and run on a 12% SDS-PAGE gel. The SDS-PAGE gels were scanned with a generic scanner and minimally processed. Cleavage assays were performed on Wild Type APPTM substrate for both MAMRE50 and MCMJR1 enzymes, and a control intramembrane protease.

### APPTM expression and purification

The pETM41-APPTM plasmid was transformed into *E. coli* BL21 DE3 cells and grown at 37 °C overnight on agar plates containing kanamycin. ^1^H-^15^N Wild Type and V44M-APPTM substrate was then expressed and purified using the protocol in *Chen et al*.^24^

### Solution NMR sample preparation

Following purification both enzyme and substrate were buffer exchanged into NMR buffer (25 mM sodium phosphate pH 7.2) where dodecylphosphocholine (*Anatrace:F308*) concentration was adjusted to 5% for APPTM and 0.1% for presenilin homolog. Prior to running solution NMR, D_2_O was added into samples to a final concentration of 10%.

### PSH cleavage of APPTM in NMR sample

One hour 2D ^1^H-^15^N TROSY experiments were collected at 40 °C on an 800 MHz Bruker spectrometer equipped with a cryoprobe. MAMRE50 and MCMJR1 enzymes were added to ^15^N-labeled V44M-APPTM samples at a ratio of 1:1. 2D TROSY experiments were collected for ^15^N V44M-APPTM alone and at timepoints of 0, 12 and 24 hours after the addition of enzyme.

### Titration of PSH into APPTM NMR sample

1D and 2D ^1^H-^15^N 2D TROSY experiments were collected at 40 °C on an 800 MHz Bruker spectrometer equipped with a cryoprobe. The number of scans collected were adjusted to account for dilution and cleavage of the substrate over time. Ten total titration points were collected ranging from 0:1 to 15:1 MCMJR1 to substrate. Titrations were collected for both ^15^N V44M-APPTM and ^15^N Wild Type-APPTM in the presence of MCMJR1 enzyme.

### Data conversion, processing and analysis

All data was converted and processed in nmrDraw and analyzed in Sparky and Microsoft Excel. Combined nitrogen and hydrogen chemical shift perturbations were calculated using:

1 $$CSP=\sqrt{{(10{\rm{\Delta }}{\delta }_{H})}^{2}+{({\rm{\Delta }}{\delta }_{N})}^{2}}$$

where Δ*δ*_*H*_ and Δ*δ*_*N*_ define the change in chemical shift in the hydrogen and nitrogen dimensions from apo APPTM to PSH-tritrated APPTM, respectively.

### Dataset availability statement

The datasets generated during and/or analysed during the current study are available from the corresponding author on reasonable request.
